# Unusual *Cryptosporidium* Genotypes in Human Cases of Diarrhea

**DOI:** 10.3201/eid1411.080239

**Published:** 2008-11

**Authors:** Guy Robinson, Kristin Elwin, Rachel M. Chalmers

**Affiliations:** UK Cryptosporidium Reference Unit, Swansea, UK

**Keywords:** Cryptosporidium, genotype, human, diarrhea, SSU rRNA, HSP70, COWP, dispatch

## Abstract

Several *Cryptosporidium* spp. are known to infect humans, but most cases of illness are caused by *Cryptosporidium hominis* or *C. parvum*. During a long-term genotyping in the United Kingdom, we identified 3 unusual *Cryptosporidium* genotypes (skunk, horse, and rabbit) in human patients with diarrhea.

*Cryptosporidium* spp. are frequently a cause of diarrheal disease in immunocompetent as well as immunocompromised humans. Over the past decade molecular methods have enabled the characterization and identification of species and genotypes within the genus. The taxonomy is under continual review, but so far 20 valid species and numerous genotypes have been described. Many are named after the original host from which the isolate was recovered and are often referred to as “host-adapted” (*1**,**2*). Most human infections are caused by *C. hominis* or *C*. *parvum* but *C*. *meleagridis*, *C*. *felis*, *C*. *canis*, *C*. *suis*, *C*. *muris*, *C*. *andersoni*, *C. hominis* monkey genotype, cervine genotype, and the chipmunk genotype I have also been detected (*1**–**6*). The immune status of the host is not necessarily linked to infection with other species/genotypes (*1**,**7*). We describe 3 unusual *Cryptosporidium* genotypes detected in human patients with diarrhea.

## The Study

Since 2000, the UK Cryptosporidium Reference Unit has maintained a national collection of *Cryptosporidium* oocysts (*8*). Over 16,000 *Cryptosporidium*-positive human fecal samples have been submitted by primary diagnostic laboratories and characterized by the Reference Unit to identify the infecting species. In addition to the expected *C. hominis*, *C. parvum,* and small number of *C. meleagridis*, *C. felis*, *C. canis,* and cervine genotype isolates, 3 other genotypes (skunk, horse, and rabbit) were identified in separate samples from individual patients after the onset of diarrhea in 2000 (sample W971), 2003 (sample W6863), and 2007 (sample W16103). A routinely collected minimum dataset was submitted with each sample, and further exposure data were collected for each patient from the local Consultant in Communicable Disease Control.

To prepare isolates for molecular characterization, oocysts were concentrated by saturated salt flotation, disrupted by boiling for 1 hour and the DNA purified by using a QIAamp DNA Mini Kit (QIAGEN, Valencia, CA, USA) as previously described (*9*). All 3 isolates were characterized by PCR–restriction fragment length polymorphism (RFLP) or bidirectional sequencing (GeneService Ltd., Cambridge, UK) at the small subunit (SSU) rRNA (≈800-bp product) (*10*), *Cryptosporidium* oocyst wall protein (COWP) (≈550-bp product) (*11*) and heat shock protein (HSP) 70 (≈450-bp or ≈325-bp products) (*12*) genes. Sequences were compared with GenBank submissions by using the BLAST algorithm (www.ncbi.nlm.nih.gov/Education/BLASTinfo/BLAST_algorithm.html).

 To confirm identification, phylogenetic analysis was conducted in TREECON (www.bioinformatics.psb.ugent.be/software/details/3) with other known *Cryptosporidium* spp. and genotypes by using alignments generated in ClustalX version 2.0 (ftp://ftp.ebi.ac.uk/pub/software/clustalw2) and manually edited in BioEdit version 7.0.9 (www.mbio.ncsu.edu/BioEdit/bioedit.html). All sequences generated in this study have been submitted to GenBank under accession nos. EU437411–EU437418.

At the SSU rRNA and HSP70 genes, sequence analysis confirmed that W971, W6863, and W16103 were skunk, horse, and rabbit genotypes, respectively (Table). Isolate W971 was homologous with genotype W13 found in storm water, which, in turn, is the skunk genotype (*6*). Initially, the BLAST search for isolate W6863 erroneously indicated *C. parvum* as the most probable identity at the SSU rRNA gene, but this was due to the short length (484 bp) of the only horse genotype sequence available (AY273770) for comparison. Thus, *C. parvum* isolates that spanned our whole query sequence (787 bp) were calculated to have greater identities by BLAST. However, a detailed comparison between AY273770 and W6863 showed only 2-bp differences (including 1 insertion in our sequence) compared with 7-bp differences between W6863 and *C. parvum*. W6863 was confirmed as a variant of the horse genotype by HSP70 gene sequence analysis and SSU rRNA gene phylogenetic analysis (Figure).

**Table:** Descriptive epidemiology and identification of 3 unusual *Cryptosporidium* genotypes in patients, United Kingdom*

Sample ID	Patient details and exposures 2 weeks before illness	Identification at 3 genes (similarity to GenBank isolates)		
		SSU rRNA	HSP70	COWP
W971	25-year-old woman, swam regularly in a pool, holiday in UK forest park	W13 (790/790-bp homology to AY737559)	Skunk genotype (279/279-bp homology to AY120917)	Sequence data unavailable
W6863	30-year-old woman, immunocompetent, foreign travel, swam in a pool	Horse genotype (483/485 bp, 99.6%, similarity to AY273770)	Horse genotype (389/389-bp homology to AY273774)	*C. parvum* (503/506 bp, 99.4%, similarity to DQ388390)
W16103	48-year-old woman, immunocompetent, foreign travel (Spain), contact with animals (birds)	Rabbit genotype (784/784-bp homology to AF120901)	Rabbit genotype (279/279-bp homology to AY273775)	*C. hominis* (506/506 bp to DQ388389)

*ID, identification; SSU, small subunit; HSP, heat shock protein; COWP, *Cryptosporidium* oocyst wall protein.

**Figure:**
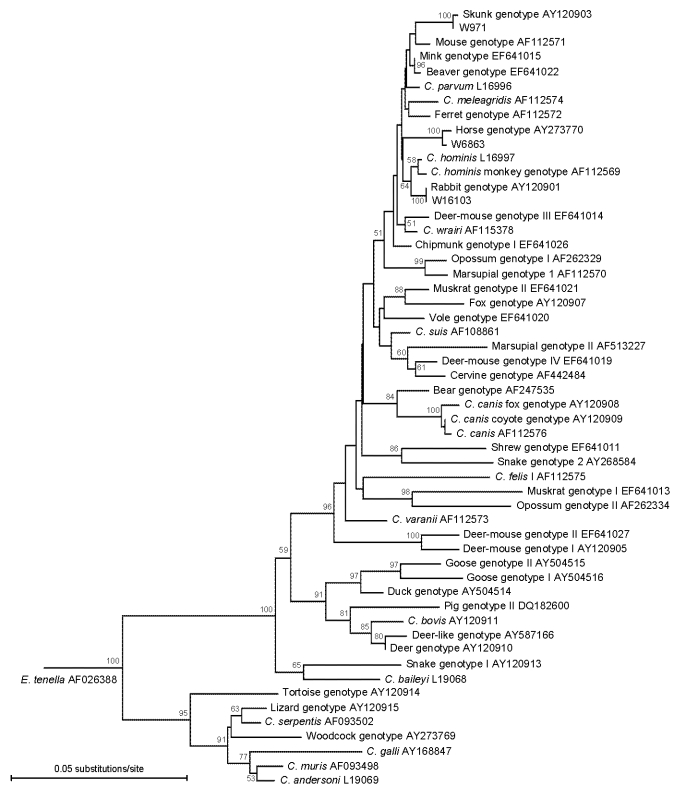
Phylogenetic relationships between 3 unusual *Cryptosporidium* genotypes and known *Cryptosporidium* species/genotypes as inferred by a neighbor-joining analysis of the small subunit rRNA gene. Evolutionary distances were calculated by the Kimura 2-parameter model with *Eimeria tenella* as an outgroup. Bootstrapping values >50% from 1,000 pseudoreplicates are shown at branches.

PCR-RFLP analysis of the SSU rRNA and COWP genes differentiated the skunk and horse genotypes from the most common human pathogens. However, identifying the rabbit genotype by PCR-RFLP at these loci was more problematic because of this genotype’s close relationship with *C. hominis*. The sequence and restriction pattern are identical at the COWP gene and, with only 4-bp substitutions (2 occurring in *SspI* cut-sites), the pattern is similar at the SSU rRNA gene. Increasing the resolution by running the agarose gel at an appropriate concentration and for as long as possible is important for the separation of the *C. hominis* diagnostic band (449 bp) from the rabbit genotype (472 bp).

## Conclusions

Information on possible risk factors was collected for the 2 weeks before the onset of illness, but we cannot be sure how these 3 persons became infected with the unusual genotypes. The skunk genotype was found in a 25-year-old woman from a rural area of southwest England, who reported no foreign travel and no contact with animals. She worked and swam regularly at an adult daycare center and had spent a week during the incubation period with clients at a holiday forest park in her region. There was no information to suggest that she was immunocompromised. The horse genotype was found in a 30-year-old immunocompetent woman also from a rural area of southwest England, who reported swimming and foreign travel (destination unknown) but no contact with animals during the incubation period. The rabbit genotype was found in a 48-year-old immunocompetent woman from a rural area of northwest England, who reported foreign travel to southern Spain and contact with wild birds (feeding ducks and geese) but no contact with other animals.

Previously, these 3 genotypes were known to cause infections only in wild or zoo animals (*13**,**14*). Wild animals are known to be an important source of *Cryptosporidium* oocysts in environmental samples and we have detected the rabbit genotype in surface waters and septic tank samples (unpub. data), but the source is unknown. Since many isolates have yet to be found in humans and although little is actually known about them, they are assumed to be insignificant to public health (*6**,**15*). The importance of unusual genotypes in humans who seek treatment for diarrheal disease warrants further investigation.
